# Microglial handling of myelin debris and remyelination after ischaemic stroke: recognition, intracellular processing, lipid fate, and oligodendroglial repair

**DOI:** 10.3389/fncel.2026.1945478

**Published:** 2026-09-15

**Authors:** Mingrui Song, Nan Bu, Long Wei, Jing Chen

**Affiliations:** 1 The Second Affiliated Hospital of Heilongjiang University of Chinese Medicine, Harbin, China; 2 Heilongjiang University of Chinese Medicine, Harbin, China

**Keywords:** ischaemic stroke, microglia, myelin debris, oligodendrocyte precursor cell, remyelination, white-matter injury

## Abstract

Post-ischaemic white-matter injury involves axonal dysfunction, myelin disorganization, delayed accumulation of myelin-derived debris, and an often incomplete regenerative response. Microglia provide a cellular link between debris clearance and white-matter repair through a sequence of functionally distinct processes encompassing selective debris recognition, internalization, intracellular degradation, lipid handling, inflammatory regulation, and oligodendroglial support. Importantly, increased myelin uptake should not be equated with successful clearance, and clearance itself does not establish effective remyelination. This review prioritizes direct evidence from adult ischaemic stroke while using studies from related white-matter and demyelination models to clarify defined cellular or metabolic steps as mechanistic context rather than as stroke-equivalent evidence. Available evidence indicates that OPC recruitment and early lineage responses are frequently preserved after stroke, whereas terminal oligodendrocyte maturation and structurally competent myelin reconstruction remain limiting steps. Within microglia, lysosomal competence and post-phagocytic lipid processing emerge as critical intermediate bottlenecks that determine whether internalized myelin is successfully disposed of or instead promotes lipid accumulation, inflammatory dysfunction, and impaired oligodendroglial support. Direct adult-stroke evidence nevertheless remains substantially more limited than the broader mechanistic literature derived from related experimental systems. We therefore propose that repair-relevant microglial activity should be evaluated across sequential checkpoints—from selective recognition and intracellular processing to lipid disposal, oligodendroglial maturation, and structurally and functionally validated remyelination.

## 1 Introduction

White-matter injury after ischaemic stroke extends beyond oligodendrocyte loss. Axonal energetic disturbance, paranodal disorganization, myelin structural failure, inflammatory activation, and cell-death programs develop on partly overlapping timescales, creating a lesion environment in which damaged myelin can persist after the initial insult (Kollai et al., 2025; Qian et al., 2025; Feng et al., 2025). In experimental stroke, oligodendrocyte death and myelin loss may remain substantial at 2 weeks, whereas new-myelin formation is still insufficient at 4 and 8 weeks (Cheng et al., 2024). This temporal separation places the handling of myelin-derived debris at the center of post-ischaemic white-matter repair rather than treating it as a secondary histological event.

Microglia can connect lesion cleanup with white-matter repair. Their contribution begins with recognition and uptake of damaged myelin, but also involves auto-phagic and lysosomal processing, cholesterol handling, inflammatory resolution, and trophic support for oligodendrocyte precursor cells (OPCs) and mature oligodendrocytes. Direct stroke studies associate TREM2-dependent microglial phagocytosis with cholesterol transfer, oligodendrocyte differentiation, and remyelination (Qin et al., 2026; Xu et al., 2023; Yu et al., 2025). By contrast, persistent myelin debris, lipid overload, and defective intracellular degradation are linked to inflammatory programs that can limit oligodendroglial maturation (Maimaiti et al., 2024; Montilla et al., 2025; Qiu et al., 2026). Throughout this review, we define uptake as cargo internalization, clearance as successful degradation and disposal of the internalized material, and remyelination as reconstruction of structurally competent myelin around axons. This distinction raises the central question addressed in this review: under what conditions does microglial uptake of post-ischaemic myelin debris progress to successful intracellular disposal and ultimately facilitate structurally and functionally meaningful remyelination?

The evidence base is heterogeneous. Adult cerebral ischaemia and experimental stroke provide the most directly relevant data, whereas chronic cerebral hypoperfusion, neonatal hypoxic-ischaemic injury, focal demyelination, multiple sclerosis, spinal cord injury, and peripheral-nerve studies primarily provide mechanistic context. Accordingly, this review treats adult ischaemic stroke as the principal evidence base and uses other settings only to clarify a defined cellular or metabolic step, such findings are not interpreted as stroke-equivalent proof. Previous reviews have established important roles for microglia in myelin-debris removal, remyelination, inflammatory regulation, cholesterol metabolism, and oligodendroglial support. Lloyd and Miron provided a broad framework for the pro-remyelinating functions of microglia across demyelinating conditions, whereas Raffaele and Fumagalli specifically examined microglial dynamics and their relationship to myelin repair after ischaemic stroke (Raffaele and Fumagalli, 2022; Lloyd and Miron, 2019). Building on these contributions, a remaining conceptual gap concerns how individual steps of debris handling are functionally connected and where this sequence fails after cerebral ischaemia. In particular, increased phagocytic uptake is frequently used as a surrogate for effective clearance, although internalization, intracellular degradation, lipid disposal, oligodendroglial maturation, and reconstruction of structurally competent myelin represent biologically distinct endpoints. The distinctive focus of the present review is therefore to treat myelin-debris handling as a multistep clearance-to-remyelination process and to evaluate the evidence supporting—or failing to support—the transition between successive checkpoints. Against this background, we organize the available evidence along a clearance-to-remyelination sequence comprising debris generation, selective recognition, cellular uptake, intracellular degradation, lipid disposal, oligodendroglial support, and structurally validated remyelination. At each step, we distinguish direct adult-stroke evidence from mechanistically informative evidence derived from related models and identify the principal biological bottlenecks that prevent progression to the next stage (Figure 1).

**Figure 1:**
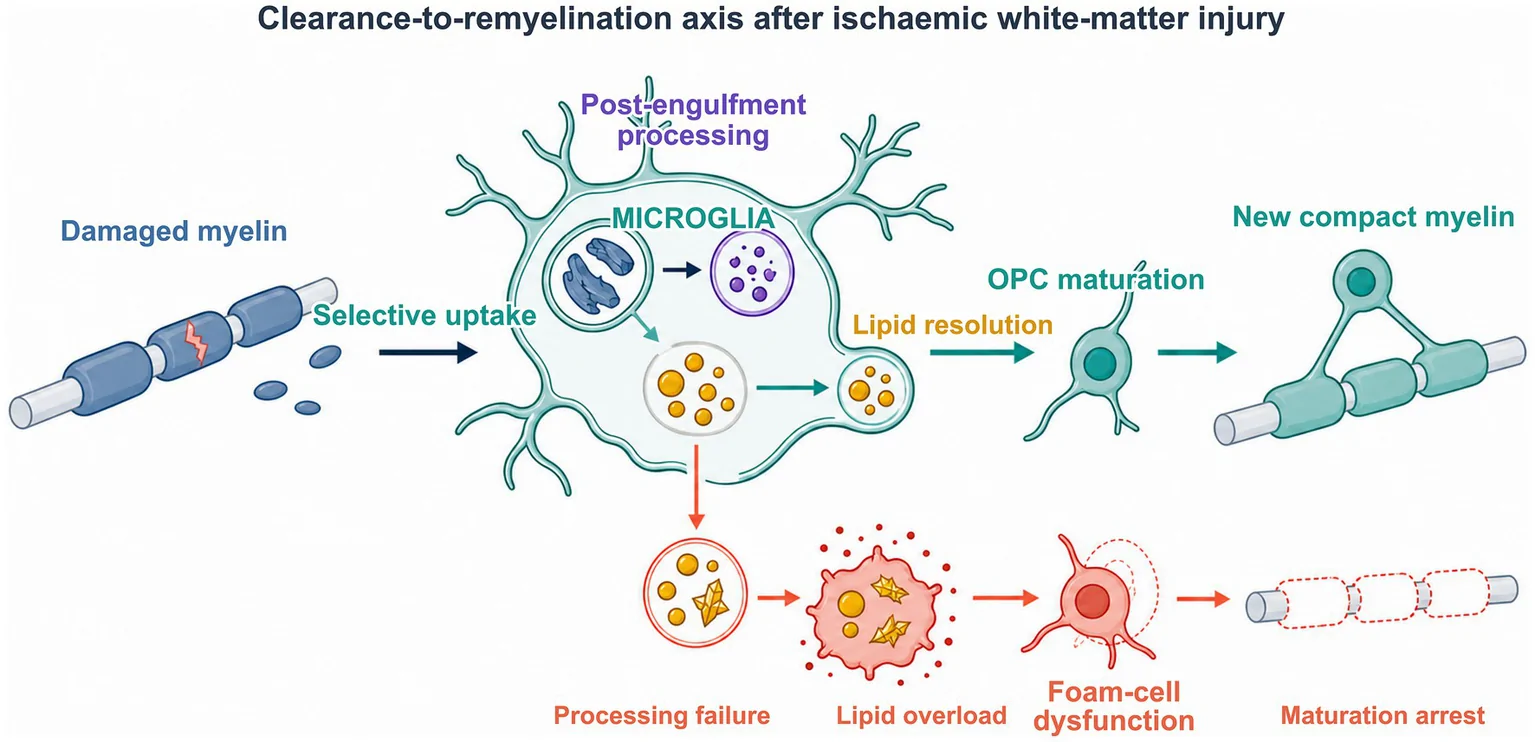
Clearance-to-remyelination axis after ischaemic white-matter injury. Ischaemic white-matter injury generates damaged myelin that must be selectively internalised and successfully processed before its lipid burden can be resolved. A repair-compatible route couples post-engulfment degradation and lipid resolution to oligodendrocyte precursor cell (OPC) maturation and formation of new compact myelin. A divergent route depicts post-engulfment processing failure, persistent lipid overload, foam-cell-like microglial dysfunction and restricted oligodendroglial maturation. The scheme is a mechanistic synthesis and does not imply that uptake alone establishes clearance or that clearance alone establishes remyelination.

### 1.1 Literature search and evidence classification

This narrative review was informed by a structured PubMed search completed on 19 June 2026. No lower publication-date limit was applied, searches were updated through 19 June 2026. Search terms were organized into three core conceptual domains: (A) ischaemic stroke or cerebral ischaemia, (B) microglia/macrophage-mediated myelin-debris recognition, phagocytosis, and intracellular handling, and (C) remyelination, oligodendrocyte-lineage repair, or white-matter repair. The primary high-specificity search combined all three domains (A AND B AND C). To capture mechanistically relevant studies in which all three concepts were not represented simultaneously, supplementary pairwise searches were performed using A AND B, A AND C, and B AND C. Additional targeted searches addressed defined stages of the clearance-to-remyelination sequence, including receptor-mediated recognition, autophagic–lysosomal processing, lipid and cholesterol handling, and OPC maturation. Representative Boolean search strategies are provided in Supplementary Table S1. Original human, *in vivo*, ex vivo, and *in vitro* studies were considered when they addressed a defined step in the clearance-to-remyelination sequence or a downstream structural or functional repair outcome. Reviews were used for background interpretation and citation tracking. Adult human and experimental ischaemic stroke studies formed the primary evidence base. Chronic cerebral hypoperfusion and neonatal hypoxic–ischaemic models were treated as ischaemia-related but non-equivalent evidence, whereas non-ischaemic demyelination, spinal-cord injury, peripheral-nerve injury, and isolated-cell models were included only when they clarified a defined mechanistic step for which direct adult-stroke evidence was insufficient. Cell-specific genetic and rescue studies were weighted more strongly than observational or marker-based findings. This was not a systematic review, and no formal risk-of-bias or quantitative synthesis was performed.

## 2 Biological basis of post-ischaemic white-matter injury and remyelination

### 2.1 Temporal and spatial evolution of secondary demyelination and remyelination

Post-ischaemic white-matter injury is best understood as an early destabilization of the axon–paranode–myelin unit rather than as isolated oligodendrocyte loss. Stroke multi-omics data associate myelin-related changes with mitochondrial respiration, calcium homeostasis, cytoskeletal regulation, apoptosis, and myelin dysfunction, supporting a multifactorial injury process rather than a single initiating mechanism (Qian et al., 2025). Mechanistic evidence from chronic cerebral hypoperfusion further places axonal mitochondrial retention and reactive oxygen species (ROS) upstream of paranodal retraction and myelin instability (Feng et al., 2025). However, inherited and experimentally induced myelin abnormalities indicate that sheath disruption can also precede overt microglial or astrocytic activation, whereas neonatal hypoxic–ischaemic and other white-matter disorders show that acute biochemical stress and later structural myelin injury may be temporally separated (Reyes-Corral et al., 2025; Pedroza-García et al., 2022; Zhang Q. et al., 2025; Bergner et al., 2021; Rajani et al., 2021; Hümmert et al., 2026). These external models clarify possible initiating routes but are not equivalent to adult stroke. Collectively, the evidence argues against a universal sequence in which oligodendrocyte death invariably precedes axonal, paranodal, and myelin disruption.

The subacute period is therefore best understood as a temporally overlapping but asynchronous state in which inflammatory activation, myelin degeneration, debris persistence, phagocyte engagement, and endogenous repair evolve on partly distinct trajectories. Human post-mortem evidence provides the most disease-relevant temporal framework: microglial activation in the corticospinal tract was detectable from day 3 after stroke, whereas histological evidence of axonal degeneration and myelin degradation emerged from day 7 (Kollai et al., 2025). Experimental cerebral ischaemia similarly induces early astrocytic proliferation followed by a later NG2-positive glial response (Steliga et al., 2023). Nevertheless, damaged or axon-deprived myelin can persist after axonal loss, indicating that substrate generation, microglial engagement, internalization, and degradation are not synchronized events (Doty et al., 2026). Findings from fetal ischaemia and non-ischaemic demyelination models support this interpretation but cannot define the stroke-specific sequence (Aires et al., 2022; Barletta et al., 2023; Zhou K. et al., 2022; Hahn et al., 2023). The subacute phase is therefore characterized by temporal dissociation among inflammatory activation, substrate generation, and phagocyte engagement.

Spatial heterogeneity further determines whether injured white matter progresses towards persistent demyelination or repair. Secondary abnormalities may occur within the infarct, in peri-infarct tissue, and along remote connected tracts. After distal middle cerebral artery occlusion (MCAO), contralateral corpus-callosum demyelination was associated with astrocyte-derived lipocalin-2, whereas persistent microglial accumulation and volume loss in the corpus callosum, internal capsule, and anterior commissure remained detectable more than 100 days after stroke (Huang et al., 2025; Sampath et al., 2025). Even within the same infarct, regions with comparable subacute demyelination can diverge towards chronic demyelination or near-normal recovery (Khodanovich et al., 2021). Local extracellular-matrix and vascular cues may contribute to this divergence: fibronectin can support phagocytic removal of myelin debris in the lesion core, whereas peri-infarct laminin α2 favors OPC differentiation (Shibahara et al., 2023). Accordingly, the lesion core, peri-infarct zone, contralateral white matter, and connected tracts should be regarded as biologically distinct repair niches rather than interchangeable sampling regions.

Post-ischaemic white-matter repair follows divergent temporal and spatial trajectories. Adult stroke models show prolonged deficits in new-myelin generation, whereas more permissive lesions permit progression from OPC recruitment to pre-myelinating and remyelinating oligodendrocytes (Cheng et al., 2024; Martín-Lopez et al., 2024). Macromolecular proton fraction (MPF) mapping further distinguishes infarct regions that remain chronically demyelinated from regions that recover towards normal myelin values (Khodanovich et al., 2021). These findings identify lesion location and temporal trajectory as major determinants of whether endogenous oligodendroglial responses progress towards reconstruction (Figure 2).

**Figure 2:**
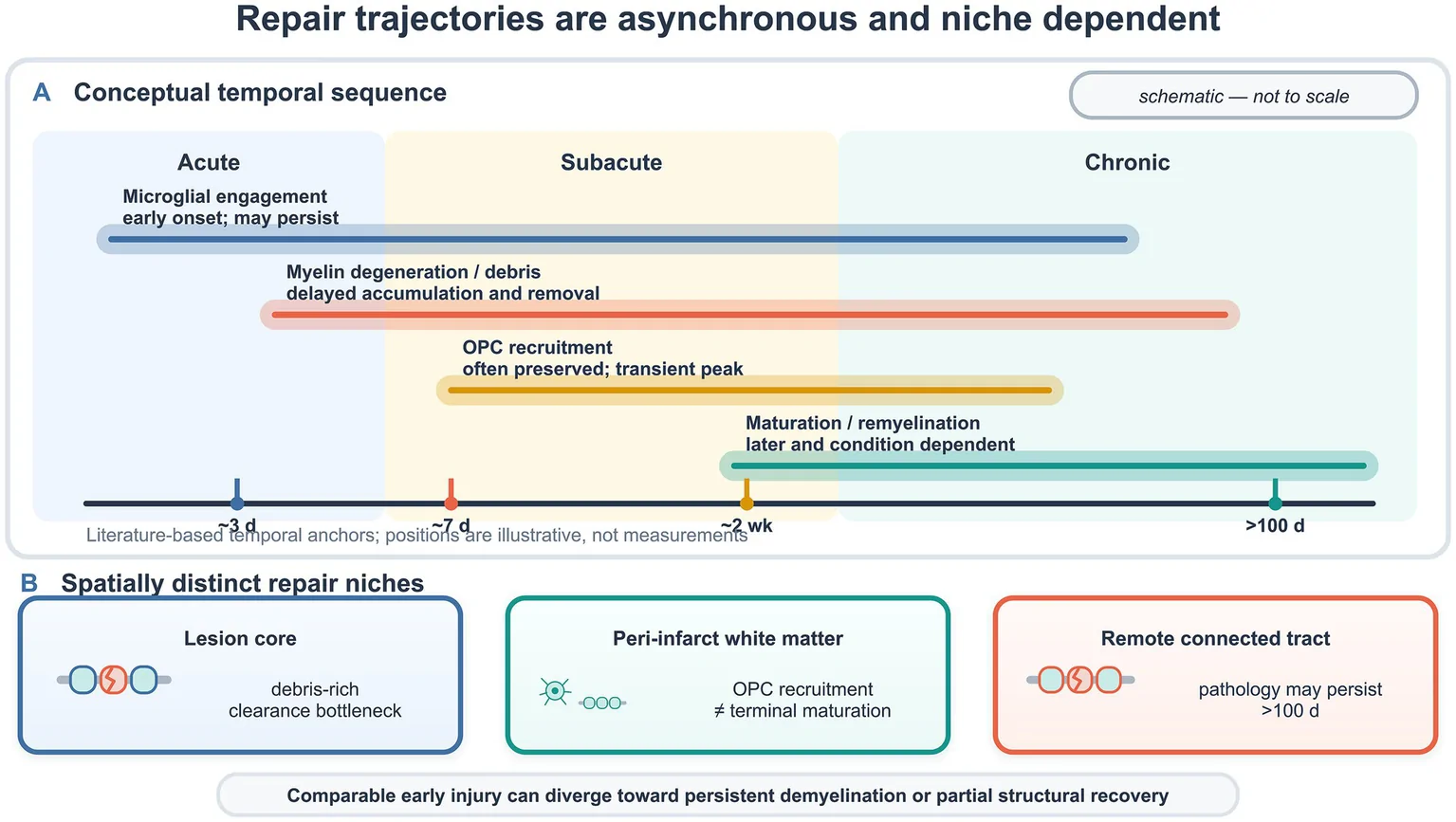
Asynchronous temporal sequence and spatially distinct repair niches after ischaemic white-matter injury **(A)** Microglial engagement, myelin degeneration/debris accumulation, OPC recruitment and maturation/remyelination are shown as overlapping conceptual intervals. The placements and durations are schematic, are not quantitatively scaled and must not be interpreted as fitted trajectories. Approximate literature-based anchors indicate early microglial responses (~3 days), prominent myelin degeneration (~7 days), experimental OPC recruitment around the second week and persistent remote white-matter pathology beyond 100 days **(B)** Lesion core, peri-infarct white matter and remote connected tracts represent distinct repair niches. Comparable early injury can diverge toward persistent demyelination or partial structural recovery.

### 2.2 OPC and oligodendrocyte-lineage responses: recruitment, maturation arrest, and myelin reconstruction

Post-ischaemic oligodendroglial repair is frequently constrained at the transition from recruited OPCs to mature, myelinating oligodendrocytes. Human pathological evidence provides the most disease-relevant support for this distinction. Peri-infarct cortex and white matter from stroke cases contained increased numbers of OLIG2-positive cells and early-stage BCAS1-positive cells with immature morphology, yet no corresponding increase in late-stage BCAS1-positive cells with mature morphology, together with reduced MBP expression (Jiang et al., 2023). GPR17-positive, BCAS1-labelled differentiation-committed OPCs also accumulated in peri-infarct regions at later stages after human ischaemic stroke (Raffaele et al., 2025). These findings indicate that recruitment and lineage commitment may persist despite insufficient progression to mature myelinating oligodendrocytes.

#### 2.2.1 OPC recruitment and proliferation

Experimental stroke studies likewise indicate that precursor recruitment remains active after ischaemic injury. Following transient MCAO, peri-infarct NG2/Ki67-positive cells showed a proliferative response that peaked during the second post-stroke week (Steliga et al., 2023). In white-matter stroke, oligodendrocyte-derived HMGB1 promoted OPC migration through TLR2-dependent signalling (Choi et al., 2023), while SHH- and NRF2-associated pathways have been linked to OPC generation or early lineage progression across stroke and related ischaemic models (Li Q. et al., 2022; Nguyen et al., 2021; Zhao H. et al., 2022; Kong et al., 2023). Collectively, these findings indicate that the injured brain retains substantial capacity for OPC recruitment and early differentiation; however, enhancement of these upstream responses does not by itself establish durable structural remyelination.

#### 2.2.2 Differentiation and terminal maturation arrest

Post-ischaemic remyelination may fail despite a robust OPC response because recruitment and terminal lineage progression are separable processes. Several experimental interventions support this dissociation. Agomelatine enhanced OPC differentiation and remyelination without increasing precursor proliferation (Wang S. et al., 2024), whereas miR-219 promoted downstream lineage progression in MCAO/reperfusion with ultrastructural evidence of improved myelin (Li W. et al., 2026). EPHA4 provides direct stroke-specific evidence for this dissociation. EphA4 signalling increased OPC proliferation while reducing mature oligodendrocyte abundance and myelin-associated proteins, whereas OPC-specific Epha4 deletion improved myelin-related outcomes and functional recovery (Liu et al., 2022). Thus, increased precursor abundance can coexist with impaired terminal maturation.

Additional pathways appear to converge on the same maturation bottleneck rather than defining independent repair programmes. Notch activation, LINGO-1/Nogo-receptor signalling, and related inhibitory programmes can restrain oligodendroglial progression or white-matter reconstruction after ischaemic injury (Zhuang et al., 2025; Strecker et al., 2025; Zhao et al., 2021; Ye et al., 2023), while non-stroke evidence involving Sox8 further supports the importance of lineage-intrinsic differentiation competence (Freudenstein et al., 2023). Together, these findings indicate that the major post-recruitment limitation lies not in OPC availability alone, but in whether recruited cells can overcome local inhibitory signals and acquire a mature myelinating phenotype.

#### 2.2.3 Myelin reconstruction versus expression of myelin markers

Increased oligodendroglial cell numbers or myelin-associated protein expression do not by themselves establish successful myelin reconstruction. In neonatal MCAO, expansion of OLIG2-positive EdU-labelled cells was dominated by immature oligodendroglia and was not followed by a corresponding increase in mature labelled cells, while myelinated axons remained reduced (Frazier et al., 2023). Similarly, after neonatal hypoxic–ischaemic injury, increased oligodendrocyte proliferation and myelin-protein expression coexisted with persistent hypomyelination and severe sheath delamination (Janowska et al., 2024). Although these models are not equivalent to adult stroke, they illustrate why structural validation is required before marker changes are interpreted as remyelination. Accordingly, claims of remyelination should require evidence of mature oligodendrocyte generation together with structural myelin reconstruction, rather than relying on OPC abundance or myelin-protein expression alone.

#### 2.2.4 Metabolic constraints on oligodendroglial maturation

Metabolic resilience is an additional prerequisite for lineage progression. Mitochondrial transfer into OPCs improved mitochondrial function, survival, proliferation, MBP expression, and the number of normally myelinated axons after focal cerebral ischaemia (Chen T. et al., 2022). By contrast, repeated ischaemia stabilised BNIP3 and increased OPC vulnerability, whereas endoplasmic-reticulum stress, mitochondrial dysfunction, oxidative stress, excitotoxicity, ionic dysregulation, and ferroptosis can each constrain oligodendroglial survival or maturation (Guan et al., 2022; Zhang L. et al., 2025; Hong et al., 2023; Yang et al., 2025; Gao et al., 2026). Together, these observations identify terminal maturation and metabolic competence as major downstream bottlenecks after stroke, while the contribution of precursor generation remains lesion- and stage-dependent. This distinction provides the rationale for the clearance-centered analysis below.

## 3 Microglial myelin-debris clearance: cellular sources, recognition, and intracellular processing

### 3.1 Cellular sources, temporal transitions, and the balance between reparative and injurious phagocytosis

The amount of myelin internalized is not sufficient to define repair-relevant phagocytosis; phagocyte identity and downstream functional competence also matter. Resident microglia and monocyte-derived macrophages can both remove myelin debris, but they are not functionally interchangeable. In demyelinating lesions, macrophages increased debris uptake after microglial depletion, yet this compensation did not prevent reductions in OPC recruitment, proliferation, differentiation, and remyelination (Baaklini et al., 2023). Consistently, comparative cortical and spinal ischaemic lesions showed that greater microglial representation was associated with more effective remyelination, whereas microglial—but not haematogenous macrophage—depletion impaired remyelination (Pavic et al., 2021). Together, these findings indicate that compensatory debris uptake by infiltrating macrophages does not necessarily constitute functional replacement of resident microglia. Repair outcome therefore depends not only on how much debris is internalised, but also on which phagocyte performs the uptake and whether that cell can couple phagocytosis to downstream repair-supportive functions.

Because debris generation and phagocyte engagement are temporally dissociated after stroke, as outlined in Section 2.1, interventions should be matched to the evolving substrate burden and the processing capacity of participating phagocytes. Acute SMO-dependent restraint of microglial activation further supports phase-specific regulation of phagocyte state (Liao et al., 2023).

Target selectivity constitutes a third determinant of phagocytic outcome, because damaged sheaths, intact myelin, and oligodendrocyte somata are recognised through partly distinct cues (Gitik et al., 2023; Peterson et al., 2021; Wang et al., 2021; Wang K. et al., 2022; Olveda et al., 2024).

The balance between beneficial and harmful phagocytosis is therefore determined by phagocyte identity, lesion phase, target selectivity, and downstream processing competence. High debris uptake alone should not be interpreted as repair-relevant clearance, because internalization may fail to progress to successful disposal or may occur in a cellular state that does not support subsequent oligodendroglial repair.

### 3.2 Myelin recognition, phagocytic checkpoints, and receptor–signal networks

Myelin debris is not a passive substrate. Its entry into the clearance pathway is governed by competing pro- and anti-phagocytic cues rather than by phagocyte activation alone (Chen W. et al., 2022). Conceptually, this process can be divided into four functional checkpoints: opsonisation and “eat-me” signaling, inhibitory “do not-eat-me” signaling, engagement of engulfment receptors, and intracellular signaling that determines whether receptor activation produces a competent phagocytic program.

#### 3.2.1 Opsonisation and inhibitory recognition checkpoints

Selective entry of damaged myelin into the phagocytic pathway reflects the balance between pro- and anti-phagocytic recognition. Complement-mediated opsonisation provides an “eat-me” mechanism: C1q opsonisation promotes CR3-dependent uptake of growth-inhibitory myelin debris, whereas disruption of the C1q/C3/CR3 axis reduces debris-associated regenerative responses (Peterson et al., 2021). In contrast, myelin CD47 engages SIRPα to restrain phagocytosis, and Cd47 deletion can accelerate debris removal (Gitik et al., 2023). Importantly, however, CD47 blockade does not uniformly improve repair, as antibody-mediated inhibition impaired the emergence of phagocytic microglia and remyelination in EAE (Wang et al., 2021). Additional studies show that inflammatory or intracellular state can modify substrate selectivity (Wang K. et al., 2022; Standiford et al., 2021). Thus, repair-relevant recognition depends on selective targeting of damaged myelin rather than indiscriminate enhancement of phagocytosis (Brown et al., 2023; DeVries et al., 2024; Standiford et al., 2021).

#### 3.2.2 TREM2 as a context-dependent regulator of phagocytic competence

TREM2 functions as a context-dependent regulator of phagocytic competence rather than as a uniformly pro-remyelinating receptor. In focal demyelination, Trem2 deficiency impaired microglial migration and myelin-debris uptake and delayed remyelination (Wang et al., 2023a), while an NMOSD model showed deficits in both debris uptake and degradation together with impaired oligodendrocyte proliferation and maturation (You et al., 2023). More directly relevant stroke studies associate TREM2-dependent microglial activity with myelin phagocytosis, cholesterol transfer, oligodendrocyte differentiation, and remyelination (Qin et al., 2026; Xu et al., 2023; Yu et al., 2025).

The repair consequence of TREM2 nevertheless depends on receptor integrity, injury phase, and the lipid-processing state of the responding cell. Studies of receptor shedding and demyelinating lesions indicate that altered TREM2 availability or processing can modify phagocytic and remyelinating responses (Beckmann et al., 2023; Chen et al., 2026; Su et al., 2026; Aguirre Candia et al., 2026; Hou et al., 2025; Gouna et al., 2021; McCray et al., 2024; Li Z. et al., 2024), whereas Trem2 deletion during chronic cerebral hypoperfusion reduced both phagocytosis and lipid-droplet accumulation but also decreased white-matter injury (Pang et al., 2023). Taken together, these findings indicate that TREM2 regulates phagocytic–metabolic competence, but its ultimate effect depends on whether downstream lipid handling and inflammatory resolution remain compatible with repair.

#### 3.2.3 Engulfment and scavenger-receptor networks

Beyond TREM2, debris uptake is supported by partially redundant bridging and scavenger-receptor systems. In chronic cerebral hypoperfusion, astrocyte-derived MFG-E8 enhanced microglial uptake through αVβ3/αVβ5–RAC1 signalling and increased IGF-1 production and mature oligodendrocytes (Dong et al., 2024). After MCAO, Gas6 accelerated microglial debris clearance and cholesterol-transport programmes, whereas disruption of AXL reduced early phagocytic competence and promoted later lipid accumulation (Jia et al., 2026; Jia et al., 2024). In experimental ischaemic stroke, MSR1 deficiency aggravated white-matter injury, whereas MSR1 overexpression accelerated myelin-debris clearance (Wei et al., 2024). MERTK- and CD36-associated studies further support the existence of a broader, state-dependent scavenger network (Zheng et al., 2022; Nguyen et al., 2023; Luo et al., 2025; Nakagomi et al., 2024; Sun et al., 2023; Yu et al., 2026). Together, these observations argue against a single dominant recognition receptor, productive uptake emerges from coordinated receptor systems whose output depends on the inflammatory and metabolic state of the phagocyte (Brousse et al., 2021; Dong et al., 2024; Xia et al., 2025; Wei et al., 2024; Luo et al., 2025; Nakagomi et al., 2024; Sun et al., 2023; Yu et al., 2026). Intracellular signaling controlling phagocytic competence

Downstream signaling determines whether receptor engagement becomes productive clearance. In chronic ischaemic white-matter injury, ADORA3 antagonism increased myelin-debris phagocytosis through cAMP/PKA/p-CREB signaling (Xu Y. et al., 2024), providing ischaemia-related evidence for regulation of uptake competence. More directly, cGAS-STING activation after tMCAO impaired both myelin-debris uptake and degradation and hindered oligodendrocyte differentiation and maturation (Maimaiti et al., 2024; Zhu et al., 2023), demonstrating that a single inflammatory program can disrupt multiple checkpoints of the clearance-to-remyelination sequence. Mechanistic studies in non-stroke models involving AhR/SYK, PLD4/AKT, and TNF/IL-1 signalling support the same general principle (Wang et al., 2023b; Sun et al., 2024; Boutou et al., 2024). Thus, productive myelin clearance emerges from a combinatorial recognition network in which downstream signaling acts as a functional gate between receptor engagement, competent uptake, intracellular processing, and a repair-compatible phagocyte state; its output therefore depends on ligand context, receptor integrity, injury phase, and downstream processing competence (Figure 3).

**Figure 3:**
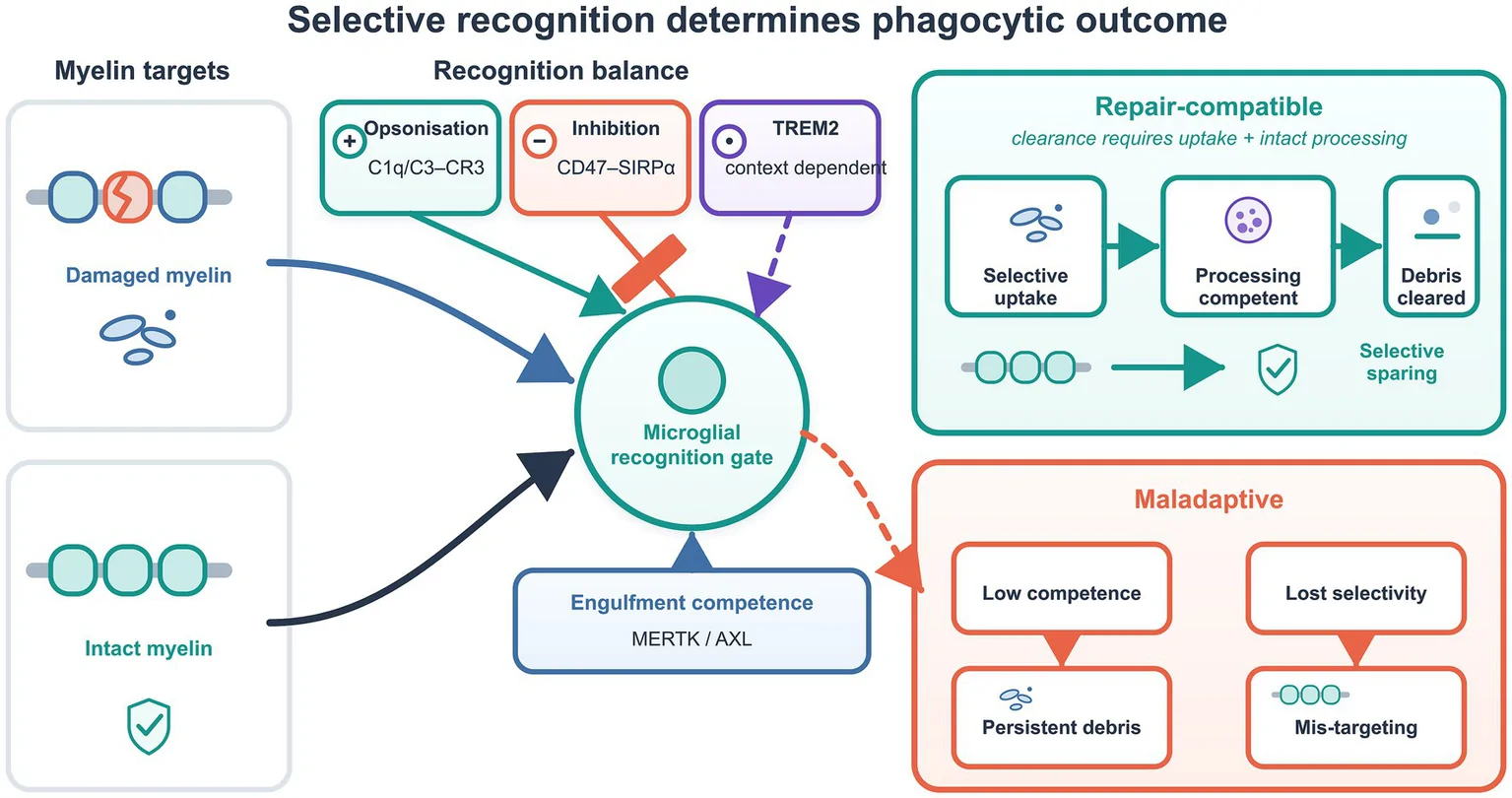
Selective myelin recognition determines phagocytic outcome. Entry into the phagocytic pathway reflects the balance between representative pro-phagocytic, inhibitory and context-dependent inputs. Complement opsonisation through C1q/C3–CR3 promotes recognition of damaged myelin, whereas CD47–SIRPα provides an inhibitory checkpoint. TREM2 is shown as context dependent, and MERTK/AXL represents engulfment competence. Repair-compatible clearance requires selective uptake of damaged myelin together with intact post-engulfment processing, while intact myelin is spared. Reduced competence can leave debris persistent, whereas loss of selectivity can promote inappropriate targeting.

### 3.3 Post-engulfment processing: why engulfment does not guarantee clearance

#### 3.3.1 Distinguishing cargo entry from post-engulfment failure

Engulfment and clearance represent mechanistically distinct stages of myelin-debris handling. After cargo entry, successful disposal requires coordinated phagosomal maturation, autophagic processing, and lysosomal degradation, each of which may become limiting despite preserved initial uptake (Hammel et al., 2022). Direct stroke evidence further shows that phagocytic dysfunction can involve both impaired engulfment and defective degradation and is associated with oxygen- and nutrient-deprivation-induced energy depletion, reduced microglial process motility, and lysosomal exhaustion (Beccari et al., 2023). Importantly, these defects need not occur in parallel. Failure can arise at distinct stages: debris may fail to enter the cell, internalized cargo may fail to progress through phagosomal or autophagic processing, or lysosomal degradation may become insufficient despite preserved uptake. This distinction is particularly well illustrated by IRF5-deficient microglia, in which myelin phagocytosis remains relatively preserved whereas intracellular degradation is insufficient, resulting in accumulation of lipid droplets, cholesterol esters, and cholesterol crystals and impaired remyelination (Montilla et al., 2025). IRF5 deficiency therefore represents a selective post-engulfment failure rather than a primary defect in cargo entry. By contrast, Trem2 deficiency suppressed both myelin-debris uptake and degradation in an NMOSD model (You et al., 2023), indicating a broader defect affecting cargo entry and downstream processing simultaneously.

#### 3.3.2 Autophagic and phagosomal processing

Successful post-engulfment processing requires preservation of autophagic competence and completion of degradative flux rather than simply increased autophagy initiation. Direct stroke evidence shows that oxygen and nutrient deprivation can simultaneously impair microglial engulfment and degradation, while basal autophagy is required to maintain cell survival and phagocytic competence (Beccari et al., 2023). Similarly, ULK1 deletion after photothrombotic ischaemia reduced microglial myelin-debris uptake and increased debris accumulation within the infarct (Xiong et al., 2025).

Evidence from demyelinating and traumatic models extends this principle to downstream flux: impaired autophagosome–lysosome fusion, reduced autophagic competence, or pyroptosis-associated disruption of autophagy can limit myelin processing and remyelination (Yan et al., 2026a; Wu et al., 2022; Wang D. et al., 2025). LC3-associated phagocytosis further indicates that shared autophagic machinery can be allocated differently according to cargo and phagocyte state (Cai et al., 2023), while alternative cellular routes reported outside stroke provide contextual evidence that myelin disposal is not restricted to a single canonical macroautophagic pathway (Jo et al., 2023; Weiß et al., 2023; Wu et al., 2024). Thus, the relevant failure mode is incomplete processing flux rather than simply low or high autophagy activity: repair-relevant clearance requires autophagic machinery to remain functionally available and capable of completing cargo processing under injury-specific metabolic and inflammatory constraints.

#### 3.3.3 Lysosomal disposal and degradative capacity

Once internalized myelin reaches the lysosomal compartment, successful disposal depends on adequate acidification, enzymatic activity, organelle integrity, and ionic homeostasis. Direct white-matter stroke evidence identifies lysosomal iron accumulation as one such failure mode. SLC11A1-dependent iron loading persisted from the acute to subacute phase and impaired both myelin-debris uptake and degradation; iron chelation, microglia-specific SLC11A1 knockdown, or pharmacological antagonism reduced this burden and improved clearance (Qiu et al., 2026). Mechanistic studies outside adult stroke indicate that reduced acidification, impaired enzyme activity, membrane instability, or defective lysosomal renewal can produce the same downstream phenotype, as illustrated by V-ATPase-, TMEM106B-, and TIM-3/TFEB-associated models (Li Y. et al., 2024; Zhang T. et al., 2023; Zhang X. et al., 2026).

Lysosomal failure can subsequently sustain inflammatory signaling and weaken oligodendroglial support. Dicer1 deficiency, cGAS-STING activation, and impaired TREM2/AhR-associated programmes link defective debris processing to inflammatory persistence and delayed oligodendroglial repair (Maimaiti et al., 2024; You et al., 2023; Wang et al., 2023b; Tripathi et al., 2025). Thus, lysosomal competence—not cargo uptake alone—constitutes a central post-engulfment bottleneck linking intracellular debris disposal to the repair environment required for remyelination (Figure 4).

**Figure 4:**
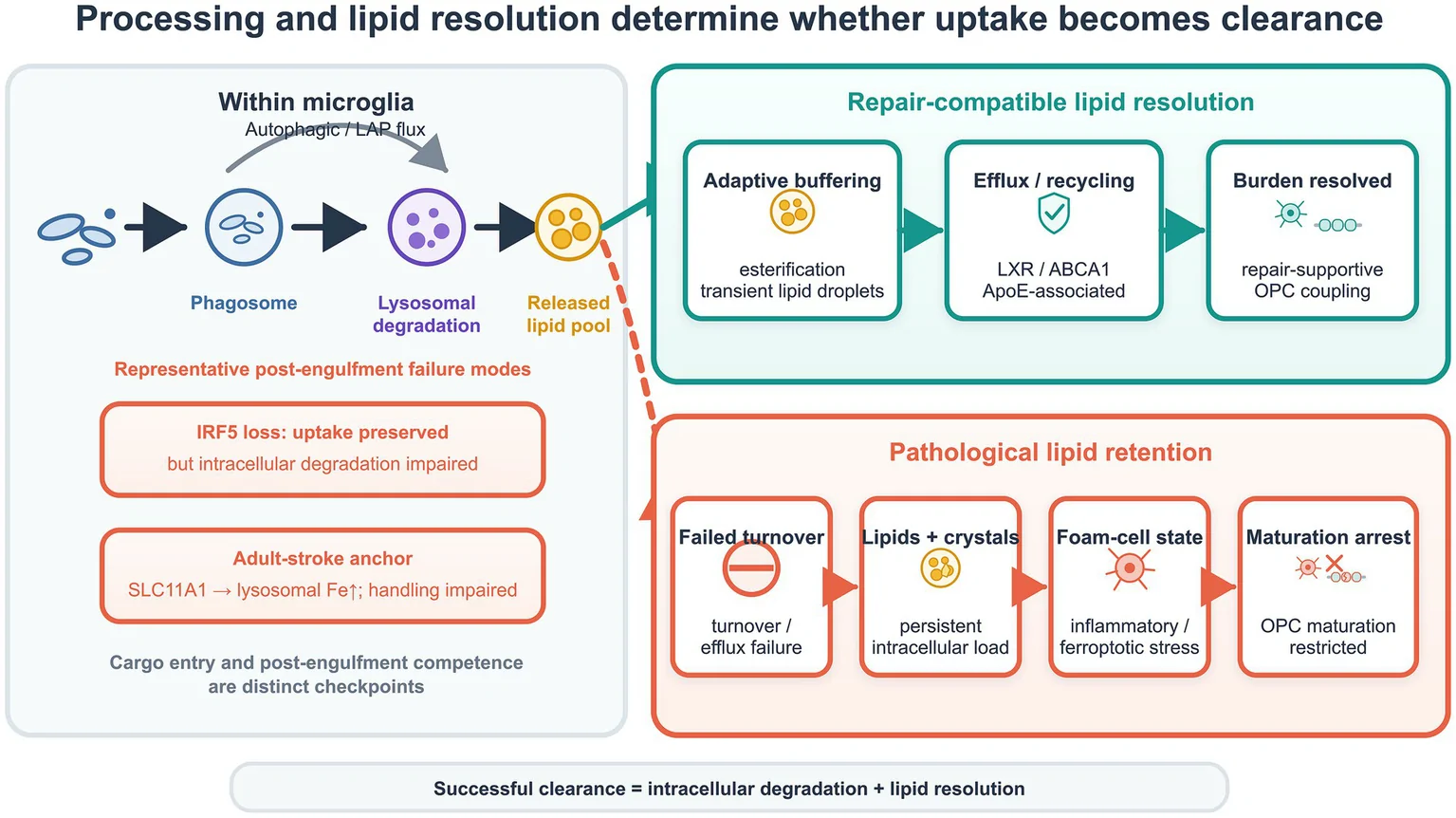
Post-engulfment processing and divergent lipid fates in microglia. Internalised myelin must progress through phagosomal, autophagic/LC3-associated phagocytic and lysosomal processing before released lipids can be resolved. IRF5 deficiency illustrates preserved uptake with impaired degradation, whereas adult white-matter stroke evidence identifies SLC11A1-associated lysosomal iron loading as a mechanism that impairs debris handling. Transient esterification and lipid-droplet buffering can remain repair compatible when followed by LXR/ABCA1- and ApoE-associated mobilisation, efflux or recycling. Failed turnover instead promotes persistent lipid and crystal accumulation, foam-cell-like dysfunction, inflammatory or ferroptosis-associated stress and restricted OPC maturation.

## 4 Post-phagocytic lipid fate and multicellular repair coupling

### 4.1 Myelin lipids, cholesterol, and lipid-droplet homeostasis: from buffering to foam-cell dysfunction

Post-phagocytic lipid burden reflects both myelin-derived cargo and, where blood–brain barrier (BBB) integrity is compromised, additional non-myelin lipid influx such as low-density lipoprotein (LDL) (Ryan et al., 2022; Qiu et al., 2021; Teo et al., 2023; Chen et al., 2024; Zhou et al., 2024). Cholesterol esterification and transient lipid-droplet formation provide an initial buffer for excess myelin-derived lipids. Cholesteryl esters increase during demyelination and normalize during successful brain remyelination, whereas their persistence in the spinal cord accompanies limited remyelination (De Silva Mohotti et al., 2026). TREM2-dependent lipid-droplet biogenesis can also limit endoplasmic-reticulum stress and support innate-immune resolution after myelin uptake (Gouna et al., 2021). Transient lipid storage is therefore compatible with repair when the stored cargo can subsequently be mobilized and cleared.

Persistent lipid storage emerges when intracellular turnover fails. In the cuprizone demyelination model, microglial PEX5 deletion increased lipid-droplet burden and reduced lipophagy; although lipid droplets declined during remyelination, intralysosomal crystals, lysosomal-damage markers, Apoe downregulation, defective debris clearance, and impaired remyelination persisted (Barnes-Vélez et al., 2026). Across related models, impaired lipophagy, altered PLIN2- or PICALM-dependent lipid handling, and prolonged myelin loading were similarly associated with persistent intracellular lipid, inflammatory myelin-laden phagocytes, or foam-cell-like dysfunction (Xia et al., 2025; Haidar et al., 2022; Loix et al., 2022; Kozlova et al., 2025; Ziaee et al., 2026). Failed lipid turnover can therefore separate apparent reduction in lipid-droplet abundance from genuine intracellular lipid resolution.

Resolution of the post-phagocytic cholesterol burden additionally requires effective sterol mobilization and efflux. In myelin-debris-loaded microglial cells, ACAT1/SOAT1 inhibition prevented cholesterol and cholesteryl-ester accumulation and increased ABCA1 expression through LXR-dependent signaling (Huynh et al., 2024). CYP46A1 activation promoted 24(S)-hydroxycholesterol formation and LXR-dependent cholesterol export, with associated improvements in white-matter integrity, debris clearance, and OPC remyelination in traumatic brain injury (Li L. et al., 2026). Conversely, myelin-derived lipid accumulation increased UBE3A-dependent ABCA1 ubiquitination and degradation, promoting lipid retention, an inflammatory macrophage phenotype, and impaired remyelination (Loix et al., 2025). Across additional injury models, enhancement of LXR/ABCA1-associated pathways reduced lipid droplets, cholesterol crystals, foam cells, or debris-laden phagocytes and was accompanied by improved myelin-related outcomes (Gao et al., 2023; Li J. et al., 2022; Ou et al., 2024; Zhang R. et al., 2023). Thus, successful intracellular degradation must be followed by cholesterol mobilisation and efflux if myelin-derived lipid burden is to be resolved.

When persistent lipid burden coincides with disturbed iron handling or insufficient antioxidant defence, lipid accumulation can progress to lipid-peroxidative and ferroptotic stress. In ageing human white-matter lesions, myelin-debris-laden degenerating microglia contained ferritin and PLIN2-positive lipid droplets together with lipid-peroxidation injury, mitochondrial and DNA-damage markers, and enrichment of ferroptosis-related genes (Adeniyi et al., 2023). Ischaemia-related evidence from chronic cerebral hypoperfusion further links sustained Nrf2 decline to increased lipid peroxidation and ferroptosis under high myelin-debris burden, whereas Nrf2 stimulation enhanced ferroptosis resistance and pro-regenerative myelination properties through lipid- and iron-metabolic reprogramming (Zhang H. et al., 2025). Post-phagocytic lipid fate therefore spans transient buffering, failed turnover and efflux, foam-cell-like dysfunction, and ferroptotic stress, with progressive loss of the microglial capacity to support white-matter repair.

### 4.2 Microglia–OPC and oligodendrocyte coupling: trophic, immune, and metabolic support

Beyond debris removal, resident microglia influence oligodendroglial repair through trophic, immune, and metabolic coupling. Depletion and lineage studies link microglial loss or dysfunction to impaired oligodendrogenesis, OPC differentiation, and adult myelin maintenance (Nguyen et al., 2023; Buller et al., 2023; McNamara et al., 2023; Ganz et al., 2023; Garcia-Martin et al., 2022; Cheng et al., 2023). Earlier M1/M2-based studies associated inflammatory myeloid states with reduced mature oligodendrocyte numbers and repair-associated states with enhanced maturation (Aydınlı et al., 2022; Ge and Li, 2023; Fang et al., 2025), while IL-13, conditioned-medium, and TGF-β studies further support state-dependent paracrine regulation of oligodendroglial differentiation (Chen D. et al., 2022; Tong et al., 2026; Wang D. et al., 2024; Pinto et al., 2025). These categories describe study-specific functional states rather than fixed myeloid phenotypes. In tMCAO mice, TREM2-dependent myelin-debris phagocytosis coincided with increased microglial IGF-1 secretion, oligodendrocyte proliferation, and remyelination (Qin et al., 2026). During chronic cerebral hypoperfusion, astrocyte-derived MFG-E8 similarly coupled microglial uptake to IGF-1 production and increased mature oligodendrocytes (Dong et al., 2024). CD11c-positive microglia and related phagocytosis/trophic-factor studies further link debris-handling competence with oligodendroglial support (Jia et al., 2023; Krishna et al., 2021; Miao et al., 2026; Yamanaka et al., 2023; Chen W. et al., 2025; Ding et al., 2026).

Metabolic exchange provides a second link between microglial state and oligodendroglial repair. After ischaemia/reperfusion, noggin-treated microglia altered glucose, lactate, and amino-acid availability, and their conditioned medium modified oligodendrocyte choline, formate, oxidative-phosphorylation intermediates, and glycerol metabolism (Lee et al., 2023). DHA-associated reparative microglia combined myelin-debris uptake with LXR-predominant cholesterol catabolism and efflux and enhanced OPC proliferation and differentiation (Liu et al., 2025). Microglial depletion, APOE2 replacement, and NHE1-associated transcriptomic changes further connect microglial lipid/metabolic state with oligodendrocyte metabolism, although direct metabolite transfer remains incompletely established (McNamara et al., 2023; Nolt et al., 2025; Song et al., 2024).

Conversely, inflammatory metabolic reprogramming can uncouple debris handling from terminal oligodendrocyte maturation. In neuroinflammatory glial cultures, microglia-derived nitric oxide and itaconate suppressed oligodendrocyte mitochondrial respiration and reduced myelin expression (Suhail et al., 2023). Related developmental and human disease models associate lipid-laden or metabolically impaired microglia/macrophages with reduced OPC differentiation or arrested oligodendroglial progression (Che et al., 2025; Jiang et al., 2025; Fransson et al., 2024; Pan et al., 2024). Microglia–oligodendroglial coupling therefore depends on whether debris handling is accompanied by trophic and metabolic support rather than persistent inflammatory or lipid-metabolic dysfunction.

### 4.3 Astrocytes, vascular and matrix cues, and peripheral immunity in the repair niche

Astrocytes can influence oligodendroglial maturation in opposing directions after white-matter injury. Following distal MCAO, astrocyte-derived lipocalin-2 promoted mature oligodendrocyte process degeneration and apoptosis (Huang et al., 2025). Conversely, astrocytic TRPA1–LIF signalling promoted OPC myelination, while enriched environments increased astrocytic BDNF and VEGF in association with remyelination after stroke (Kakae et al., 2023; Guo and Bi, 2024). Astrocytic regulation of oligodendroglial repair therefore varies with the signalling programme and injury context.

BBB disruption can alter microglial lipid handling by increasing exposure to circulating lipids. In NMOSD and chronic cerebral hypoperfusion, LDL entry across a leaky BBB increased myelin-debris engulfment but was accompanied by impaired lipid metabolism or accumulation of lipid-laden microglia with reduced pro-regenerative properties; LDL reduction or microglial LDLR knockdown alleviated demyelination (Chen et al., 2024; Zhou et al., 2024). Vascular and matrix cues also act directly on repair-associated cellular responses. Endothelial CCL21–oligodendroglial CCR7 signaling and the Cav-1–HSP90α axis modified oligodendroglial responses in chronic cerebral hypoperfusion (Tang et al., 2023; Zhao Y. et al., 2022), whereas, after experimental stroke, pericyte-derived fibronectin enhanced lesion-core myelin-debris phagocytosis and peri-infarct laminin α2 promoted OPC differentiation (Shibahara et al., 2023).

Peripheral immune cells can modify debris handling and white-matter repair in a lineage- and phase-dependent manner. After stroke, monocyte-derived macrophages can contribute to debris clearance and remyelination under permissive conditions (Ju et al., 2022; Li et al., 2025). Regulatory T-cell augmentation reduced early oligodendroglial cell death and improved later white-matter integrity, with Treg-derived osteopontin promoting reparative microglial activity (Yuan et al., 2023; Shi et al., 2021; Zera and Buckwalter, 2021). In contrast, ageing-associated CXCL10–CXCR3 signalling recruited pathogenic CD8+ T cells and promoted an interferon-activated, myelin-damaging microglial state (Groh et al., 2025; Kedia et al., 2024). Lesion phase, ageing, and comorbidity can therefore modify both the clearance environment and the capacity for oligodendroglial repair.

## 5 Constraints, therapeutic strategies, and research standards for the clearance-to-remyelination axis

### 5.1 Biological modifiers: timing, ageing, and comorbidity

The dominant constraints on debris handling and oligodendroglial repair change across post-ischaemic phases. In cerebral ischaemia/reperfusion, p39 deletion was associated with early neuroprotection but later neurological deterioration accompanied by demyelination and increased p35 expression (Meng et al., 2024). Microglial activation and white-matter attrition can persist beyond 100 days after stroke and remain associated with cognitive impairment (Sampath et al., 2025). Additional evidence involving prolonged TREM2 activation, glucocorticoid exposure, circadian regulation, and GPX4-associated ferroptotic responses further demonstrates phase-dependent changes in phagocyte and oligodendroglial states (Wu et al., 2026; Zalewska et al., 2021; Ghareghani et al., 2023; Thau-Zuchman et al., 2022; Xue et al., 2026; Zhuang et al., 2023). Thus, the checkpoint limiting repair is not fixed over time but shifts with the evolving cellular and metabolic state of the lesion.

Ageing remodels several checkpoints of the clearance-to-remyelination axis rather than uniformly suppressing phagocytosis. In aged rhesus cingulum, increased C1q deposition on myelin, reduced myelin-associated CD47, and greater microglial reactivity indicate an altered balance between pro- and anti-phagocytic recognition cues (DeVries et al., 2024). Phagocytic competence remains heterogeneous: aged white-matter microglia can retain myelin-recognition and engulfment capacity (Sanchez-Molina et al., 2021), whereas aged myeloid cells after stroke show reduced Cd36 expression and impaired phagocytosis (Ting et al., 2025). In human ageing white-matter lesions, debris-laden degenerating microglia accumulate ferritin and PLIN2-positive lipid droplets together with lipid-peroxidation and ferroptosis-related signatures (Adeniyi et al., 2023). CD22-, RXR-, and chromatin-associated studies further link ageing to modifiable defects in debris clearance and remyelination (Ting et al., 2025; Yan et al., 2026b; Tiwari et al., 2024), while primate data showing myelin fragments within Iba1-positive microglia and nonlinear OPC-density changes support persistence of a remodeled repair response (Zhang Y. et al., 2026).

Metabolic and vascular comorbidities can impose simultaneous constraints on debris handling and oligodendroglial maturation. In diabetic stroke, impaired cerebrovascular remodeling is accompanied by reduced oligodendrogenesis and white-matter integrity (Jiang et al., 2022), and these abnormalities can emerge during delayed rather than acute phases of injury (Song et al., 2023). Experimental studies further associate diabetic or metabolically stressed white matter with NLRP3-related microglial pyroptosis, defective phagocytosis, myelin-debris accumulation, and impaired remyelination (Xu et al., 2026; Xin et al., 2024; Wongpun et al., 2026). Epigenetic repression and inflammatory signaling can independently restrict OPC maturation or debris removal, including DNMT3B–REST–CNTN1 and TNF-α/PAD4–MyRF-associated mechanisms (Yao et al., 2025; Du et al., 2026b). The failure of isolated TNFR1 blockade to restore myelin integrity in obese mice further indicates that inflammatory, vascular, metabolic, and oligodendroglial constraints can coexist within the same repair environment (Haarslev et al., 2025).

### 5.2 Stage-targeted pharmacological, genetic, and cell-based strategies

Therapeutic strategies should be classified according to the specific checkpoint they directly modify rather than the number of pathways they influence. As summarized in Table 1, interventions can broadly target post-engulfment catabolism, the inflammatory–metabolic repair niche, or terminal oligodendrocyte-lineage maturation. Increased phagocytic uptake alone is insufficient to establish therapeutic benefit when downstream degradation, lipid disposal, or inflammatory resolution is not demonstrated. Conversely, interventions that improve white-matter outcomes without directly measuring debris processing should be considered repair-permissive rather than direct clearance therapies.

**Table 1:** Stage-targeted interventions and evidence positioning across the clearance-to-remyelination axis.

Target or processing stage	Representative approaches	Evidence context	Highest directly supported outcome	Principal limitation
Uptake without downstream control	Niacin-enhanced macrophage phagocytosis (Wuerch et al., 2024)	Non-ischaemic demyelination	Increased phagocytic uptake	Inconsistent tissue and functional benefit; not adult-stroke evidence
Post-engulfment catabolism	NRF2–RUBCN-associated and related microglial interventions (Shen et al., 2022; Huré et al., 2024; Lee et al., 2025; Geladaris et al., 2024; Gharibani et al., 2025)	Predominantly non-ischaemic demyelination	LC3-associated processing, inflammatory resolution, and selected OPC/remyelination responses	Direct adult-stroke validation remains limited
Inflammatory–metabolic niche remodeling	Soluble TNF, CB2R–NLRP3, PPAR-γ, AMPK and lipid-handling interventions (Xin et al., 2024; Thougaard et al., 2025; Xie et al., 2023; Zhou M. et al., 2022; Fang et al., 2024)	Mixed adult-stroke and ischaemia-related evidence	White-matter protection, oligodendrogenesis, or functional recovery	Complete intracellular debris-processing sequence is rarely assessed
Lineage-directed and cell-based repair	Akt/mTOR promotion, OPC-targeted *Olig2* mRNA, hiPSC-derived progenitors and pro-differentiation exosomes (Han et al., 2026; Xu et al., 2022; Du et al., 2026a; Xu J. et al., 2024)	Adult experimental stroke	Oligodendrocyte maturation, myelin reconstruction, and behavioral recovery	Long-term integration, durability, and safety remain incompletely defined
Multimodal delivery and niche support	Growth factors, hydrogels, MSCs and exosome platforms (Wang C. et al., 2025; Wu et al., 2023; Wang C. et al., 2022)	Mixed experimental stroke evidence	Combined trophic, vascular, anti-inflammatory, and oligodendrogenic effects	Broad biological effects obscure the active mechanism
Activity-dependent repair-niche modulation	Exercise and enriched environments (Xu et al., 2023; Guo and Bi, 2024; Han et al., 2024; Wang J. et al., 2024; Guo et al., 2022; Jiang et al., 2021)	Experimental stroke and chronic cerebral hypoperfusion	Reduced white-matter injury and inflammatory glial activity; enhanced trophic/oligodendroglial responses; myelin-debris clearance and remyelination in hypoperfusion	Direct evidence for enhanced post-engulfment degradation or lipid disposal and structurally validated remyelination after adult stroke remains insufficient

Evidence positioning reflects both model relevance and mechanistic completeness. Adult human stroke and adult experimental ischaemic stroke studies were prioritized. Ischaemia-related models, including chronic cerebral hypoperfusion and neonatal hypoxic–ischaemic injury, and non-ischaemic models were used only when they clarified a defined mechanistic step for which direct adult-stroke evidence was limited. AKT, protein kinase B; AMPK, AMP-activated protein kinase; CB2R, cannabinoid receptor type 2; hiPSC, human induced pluripotent stem cell; LC3, microtubule-associated protein 1A/1B light chain 3; MSC, mesenchymal stem cell; mTOR, mechanistic target of rapamycin; NLRP3, NLR family pyrin domain containing 3; NRF2, nuclear factor erythroid 2-related factor 2; OPC, oligodendrocyte precursor cell; PPAR-γ, peroxisome proliferator-activated receptor-γ; RUBCN, rubicon autophagy regulator; TNF, tumour necrosis factor.

Accordingly, the strength of evidence for an intervention should be judged by the specific stage of the clearance-to-remyelination axis that it directly modifies and by whether causal evidence links that modification to downstream myelin reconstruction.

### 5.3 Activity-dependent modulation of the repair niche

Activity-dependent interventions can modify the post-ischaemic repair niche, although current evidence resolves only selected checkpoints in the clearance-to-remyelination sequence. Exercise and enriched environments reduce white-matter injury and inflammatory glial activity while promoting trophic signaling and oligodendroglial responses across experimental cerebral ischaemia models (Guo and Bi, 2024; Han Y. et al., 2024; Wang J. et al., 2024; Guo et al., 2022). In chronic cerebral hypoperfusion, physical exercise was additionally associated with enhanced myelin-debris clearance and remyelination (Jiang et al., 2021), while TREM2-dependent microglial activity provides a mechanistic link between exercise and white-matter repair after stroke (Xu et al., 2023). However, direct evidence that exercise enhances post-engulfment lysosomal degradation or lipid disposal after adult stroke remains lacking. Current evidence therefore supports activity-dependent modulation of the repair niche, but not a direct effect on post-engulfment microglial clearance after adult stroke (Table 1).

### 5.4 Defining successful clearance and remyelination: an endpoint hierarchy

Claims of repair should be assigned to the highest biological level directly supported by the measured endpoint. Increased receptor expression or microglial activation indicates a cellular response; intracellular myelin indicates cargo uptake; disappearance of internalized debris requires evidence of degradation or disposal; increased OPC or myelin-protein markers indicate lineage or molecular responses; and true remyelination ultimately requires evidence of structurally competent new myelin. Functional restoration represents a further level requiring demonstration of tract conduction, network recovery, or sustained behavioral improvement. These endpoints should not be treated as interchangeable (Figure 5). Following neonatal hypoxic-ischaemic injury, OPC proliferation and myelin-protein overproduction coexisted with hypomyelination and severe sheath delamination, illustrating why structural validation should anchor claims of remyelination (Janowska et al., 2024). Endpoint validation should therefore integrate myelin ultrastructure with oligodendrocyte and microglial transcriptomic measures while accounting for treatment timing, administration route, and differences among injury models (Bokobza et al., 2025).

**Figure 5:**
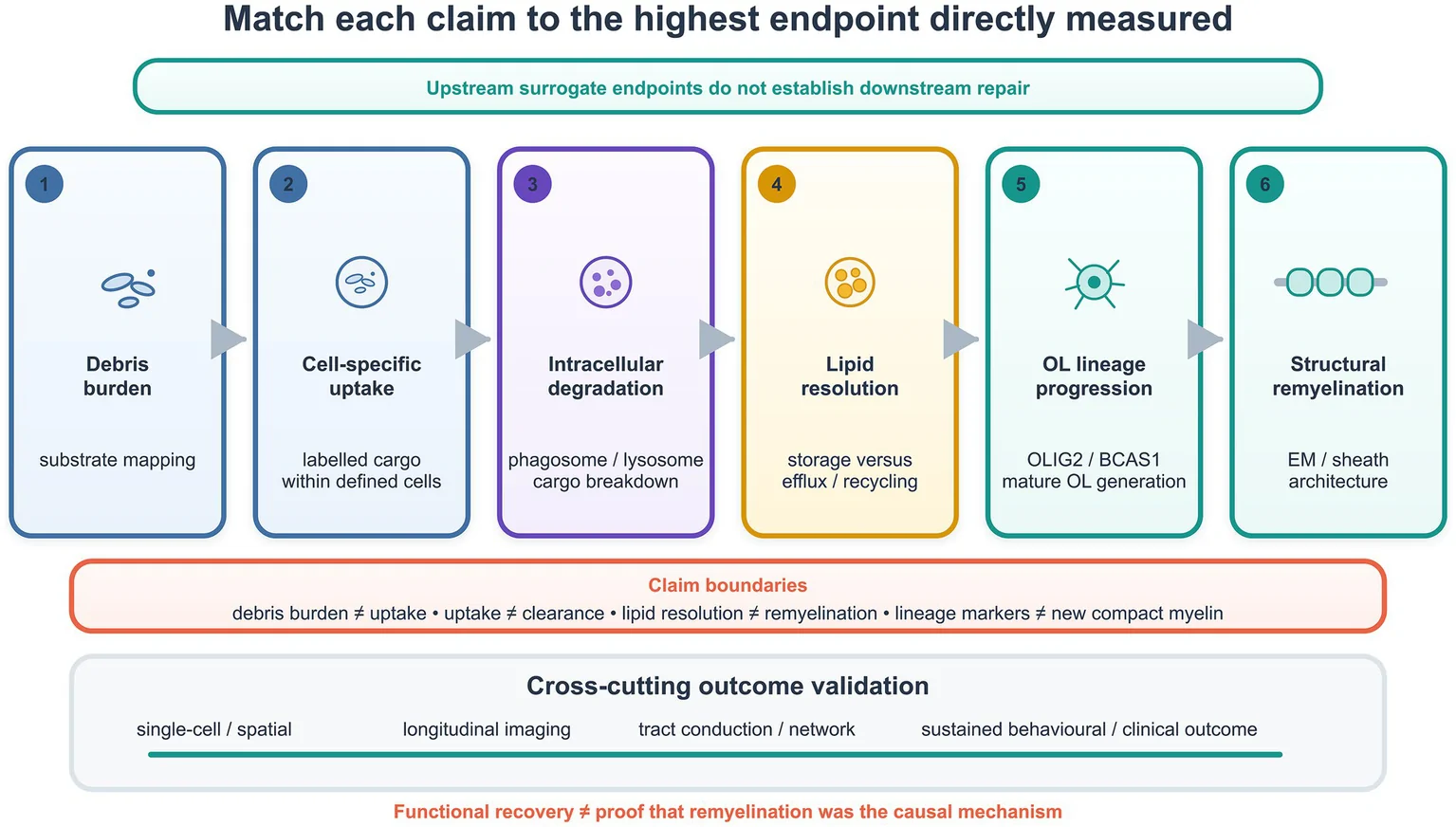
Endpoint hierarchy and claim boundaries for myelin clearance and remyelination. Experimental and clinical endpoints support different levels of inference. Substrate mapping establishes debris burden but not cell-specific uptake; intracellular cargo establishes uptake but not degradation or clearance; degradation must be accompanied by resolution of the resulting lipid burden; oligodendroglial lineage progression does not by itself establish formation of new compact myelin; and structural remyelination requires direct assessment of sheath architecture. Single-cell/spatial approaches, longitudinal imaging and tract/network or sustained clinical outcomes provide complementary validation, but functional recovery alone does not prove that remyelination was causal.

Single-cell and spatial datasets indicate that post-ischaemic clearance and remyelination involve temporally evolving, spatially restricted cell states rather than fixed marker-defined phenotypes. In hypoxic-ischaemic encephalopathy (HIE), hyperacute microglial phagocytosis preceded acute proliferation and subacute MHC II-high inflammatory microglial expansion; oligodendrocytes declined and OPCs showed Stat3-mediated ischaemic responses (Chen X. et al., 2025). In a two-vessel occlusion (2VO) model, scRNA-seq suggested four OPC subtypes with distinct differentiation pathways (Zhang et al., 2024). Although monocyte-derived macrophages (MDMs) may contribute to debris clearance while exacerbating neuroinflammation, combined scRNA-seq/scATAC-seq at 48 h after transient MCAO mapped a transition towards *Cd68*^hi^/*Ctsd*^hi^ MDMs and linked their lysosomal/lipid-processing programs to regulatory-locus accessibility (Hamblin et al., 2026). Spatial transcriptomics localized cell states to peri-infarct tissue and identified microglial/macrophage-derived galectins in LGALS9–CD44 communication; Lgals9 delivery improved long-term recovery, whereas Cd44 knockdown partially reversed these effects and inhibited oligodendrocyte differentiation and remyelination, providing functional grounds to test spatial signals causally (Han B. et al., 2024).

Human lesion analyses provide disease-relevant evidence for stage-specific oligodendroglial failure. Jiang et al. (2023) found post-mortem stroke cortex and white matter contained increased early-stage BCAS1^+^ and OLIG2^+^ cells, but no increase in late-stage BCAS1^+^ cells with mature morphology; reduced MBP accompanied this pattern, indicating insufficient OPC maturation. In contrast, small-vessel disease (SVD) lesions showed no significant increase in OLIG2^+^ or BCAS1^+^ cells, consistent with impaired OPC recruitment (Jiang et al., 2023). Zhao et al. (2026) likewise observed more OPCs and astrocytes but fewer mature oligodendrocytes in peri-infarct tissue, despite trajectories consistent with remyelination. Together, these findings distinguish insufficient OPC recruitment from post-recruitment maturation arrest.

Because MPF, myelin-water imaging, MTR, PET, and DTI report overlapping but non-identical tissue properties, each signal should be interpreted against tissue pathology and time after stroke, with imaging-based remyelination claims requiring histological validation. In experimental ischaemic stroke, MPF separated infarct regions with persistent 30–40% decreases from regions returning near-normal after one month; histology identified the former as chronically demyelinated and the latter as remyelinated (Khodanovich et al., 2021). Human longitudinal susceptibility-based imaging further associated evolving iron and myelination signals within ischaemic lesions with neurological outcomes (Uchida et al., 2024). Similarly, myelin-water imaging reflected histological differences in myelin lipids and proteins, including remyelination, whereas MTR was influenced by non-myelin components and storage duration (Wiggermann et al., 2023). Combined PET and synthetic MRI revealed an inverse association between activated microglia and myelin content, particularly in perilesional white matter (Barletta et al., 2023). DTI measures were also associated with astrocytic swelling, oligodendroglial injury, and activated microglia (Lee et al., 2021; Kor et al., 2022). Accordingly, voxelwise MRI–histology co-registration across myelin, neurofilaments, and microglial states is required before imaging can support claims of repair. Functional validation additionally requires evidence of tract conduction and distributed network performance. In cuprizone-fed mice, visual evoked potential (VEP) latency delays were associated with MBP loss in the visual pathway (Rossi et al., 2026), illustrating the value of electrophysiological readouts as a functional complement to structural myelin assessment. At the systems level, network and connectivity measures should likewise be interpreted as functional validation rather than as direct surrogates of remyelination. Structural connectivity evolves differently according to lesion size and topology, as cortical and cortico-striatal strokes generated distinct brain-wide patterns over four weeks (Mahani et al., 2026). Clinically, larger synthetic-MRI total myelin volume independently predicted good 3-month outcomes (Toko et al., 2025), whereas CSF NfL principally indexed inflammatory lesion activity and acute axonal damage in MS (van den Bosch et al., 2022). Together, longitudinal imaging, histological co-registration, tract conduction, network connectivity, and sustained behavioural outcomes provide the necessary cross-scale validation framework (Figure 5).

## 6 Discussion, limitations, and perspectives

Microglial myelin-debris clearance should be understood as a multistep biological process rather than as a single phagocytic event. Damaged myelin must first be selectively recognised and internalized, then successfully processed through phagosomal, autophagic, and lysosomal pathways, followed by appropriate handling or export of the resulting lipid burden. Only when these intracellular processes remain compatible with trophic, immune, and metabolic support for the oligodendrocyte lineage can debris removal contribute to structurally meaningful remyelination. A central implication of this framework is that increased cargo uptake is not an adequate surrogate for successful clearance. Greater intracellular myelin may indicate enhanced phagocytic activity, but it may also reflect impaired degradation or lipid disposal. The IRF5 example is particularly instructive because preserved uptake can coexist with defective degradation and intracellular cholesterol accumulation (Montilla et al., 2025). We therefore consider post-engulfment degradative and lipid-processing capacity to be a critical intermediate bottleneck linking microglial phagocytosis to repair.

A second implication concerns the oligodendrocyte lineage. Human and experimental evidence indicates that OPC recruitment and early lineage activation can persist after stroke, whereas terminal maturation and reconstruction of structurally competent myelin may remain inadequate. Consequently, increases in OPC number, differentiation-associated markers, or myelin-protein expression should not automatically be interpreted as successful remyelination.

These considerations also help reconcile apparently conflicting findings for individual pathways. TREM2, for example, is strongly associated with myelin recognition, phagocytosis, cholesterol adaptation, and remyelination, yet its effects vary with injury phase, lipid burden, receptor processing, and downstream metabolic capacity. Rather than classifying individual molecules or microglial states as uniformly beneficial or detrimental, their function should therefore be interpreted according to the specific checkpoint they regulate and whether subsequent stages of the clearance-to-remyelination sequence remain intact.

This review has several limitations. The evidence base was identified through a structured PubMed search rather than a systematic multi-database protocol, and study selection was not accompanied by formal risk-of-bias assessment. Mechanistic inferences frequently rely on heterogeneous ischaemia-related and non-ischaemic models, whereas direct adult human stroke evidence remains limited.

Evidence density also differs markedly across individual stages of the proposed sequence. Receptor signaling, phagocytic mechanisms, and intracellular metabolism are supported by a substantially larger experimental literature than the complete transition from myelin uptake to intracellular disposal and structurally validated remyelination after adult stroke. Accordingly, the proposed framework should be interpreted as an evidence-weighted mechanistic synthesis rather than as a quantitative estimate of therapeutic efficacy.

Future studies should evaluate linked stages of the clearance-to-remyelination sequence rather than isolated molecular markers. Ideally, experiments should distinguish resident microglia from infiltrating macrophages; quantify selective uptake of damaged myelin; demonstrate intracellular degradation and lipid disposal; characterize the resulting inflammatory and metabolic state; measure OPC differentiation and mature oligodendrocyte generation; and validate new myelin using ultrastructural or otherwise myelin-specific approaches. Tract conduction, network-level recovery, and sustained behavioral outcomes should then establish whether structural repair is functionally meaningful. Age, sex, metabolic comorbidity, and lesion location should be incorporated because each can alter recognition cues, lysosomal competence, lipid handling, and the repair niche.

Taken together, the available evidence supports a model in which the therapeutic value of microglial phagocytosis is determined not by the amount of myelin internalized, but by whether uptake progresses through successful intracellular processing and lipid resolution to an oligodendroglial environment capable of reconstructing structurally competent and functionally meaningful myelin. This distinction provides both a mechanistic framework for interpreting existing studies and an endpoint hierarchy for developing future strategies to promote durable white-matter repair after ischaemic stroke.
